# Urinary vanin-1, tubular injury, and graft failure in kidney transplant recipients

**DOI:** 10.1038/s41598-024-52635-x

**Published:** 2024-01-27

**Authors:** Firas F. Alkaff, Daan Kremer, Tessa M. Niekolaas, Jacob van den Born, Gerald Rimbach, Tzu-Ling Tseng, Stefan P. Berger, Stephan J. L. Bakker, Martin H. de Borst

**Affiliations:** 1 Division of Nephrology, Department of Internal Medicine, University of Groningen, University Medical Center Groningen, Hanzeplein 1, Groningen, 9713 GZ The Netherlands; 2 Division of Pharmacology and Therapy, Department of Anatomy, Histology, and Pharmacology, Faculty of Medicine Universitas Airlangga, Jl. Mayjen Prof Dr. Moestopo No 47, Surabaya, East Java 60131 Indonesia; 3 Division of Transplantation Immunology, Department of Laboratory Medicine, University of Groningen, University Medical Center Groningen, Groningen, The Netherlands; 4 Institute of Human Nutrition and Food Science, University of Kiel, Kiel, Germany; 5 Bio Preventive Medicine Corp, Hsinchu, Taiwan

**Keywords:** Biomarkers, Nephrology

## Abstract

We investigated whether urinary vascular non-inflammatory molecule-1 (vanin-1), a promising early-onset tubular injury marker, correlates with other established tubular injury markers and is associated with graft failure in kidney transplant recipients (KTR). We measured 24 h urinary vanin-1 excretion in 656 KTR (age 53 ± 13 years, 43% female, estimated glomerular filtration rate (eGFR) 53 ± 21 mL/min/1.73 m^2^) who had undergone kidney transplantation ≥ 1 year. The median 24 h urinary vanin-1 excretion was 145 [51–331] pmol/24 h. 24 h urinary vanin-1 excretion correlated weakly but significantly with other tubular injury markers (ρ = 0.14, p < 0.001 with urinary liver-type fatty acid binding protein, ρ = 0.13, p = 0.001 with urinary post-translationally modified fetuin-A protein, and ρ = 0.10, p = 0.011 with plasma neutrophil gelatinase-associated lipocalin) and with eGFR (ρ = − 0.13, p = 0.001). During a median follow-up of 7.4 [4.9–8.0] years, 94 (14%) KTR developed death-censored graft failure. In multivariable Cox regression analyses, 24 h urinary vanin-1 excretion was not associated with an increased risk of death-censored graft failure (adjusted hazard ratio [95% confidence interval] = 0.96 [0.86–1.07], p = 0.5). In conclusion, our findings do not support the role of urinary vanin-1 as a biomarker of graft failure after kidney transplantation.

## Introduction

Kidney transplantation is the preferred treatment for patients with end-stage kidney disease, as it has the lowest mortality rate and provides a better quality of life compared to other kidney replacement therapy modalities. Importantly, kidney transplantation is also the most cost-effective modality of kidney function replacement^1–3^. However, even after successful transplantation, kidney transplant recipients (KTR) remain at risk of graft failure, especially in the long term^4,5^.

Tubular injury, regardless of the underlying cause, is an important contributor to graft failure^6^. Therefore, tubular injury biomarkers could provide additional information that contributes to risk prediction after kidney transplantation beyond more established predictors focusing on the glomerulus, such as the estimated glomerular filtration rate (eGFR), which has limited predictive utility^7^. Vascular non-inflammatory molecule-1 (vanin-1) is a member of the aminohydrolase family of pantetheinases. Its physiological function is related to the pantetheinase activity, i.e., breaking down pantetheine into cysteamine (a molecule involved in redox homeostasis) and pantothenic acid (an important cofactor for Coenzyme A)^8^. Coenzyme A is a crucial molecule involved in various metabolic processes, including fatty acid synthesis and energy production. In physiological conditions, vanin-1 is expressed in many organs, with high basal expression, particularly in organs with high Coenzyme A turnover, such as liver and kidney^9,10^. Within the kidney, vanin-1 is only expressed in the brush border of proximal tubules and not in glomeruli^11,12^.

In recent years, vanin-1 has been identified as a key player in the development and continuation of certain diseases due to its enzymatic activity^8^. Furthermore, vanin-1 has been proposed as an oxidative stress sensor, as it is upregulated upon oxidative injury and involved in the regulation of redox homeostasis^10,13, 14^. Since proximal tubular epithelial cells are prone to oxidative stress^15^, and tubular injury is an important contributor to graft failure^6^, vanin-1 might be a potential candidate for an early-onset tubular injury biomarker in the post-transplant setting.

Previously, both experimental and observational studies have shown that urinary vanin-1 increased before serum creatinine or other tubular injury markers in acute kidney injury^12,16, 17^. Next to that, urinary vanin-1 was associated with eGFR and kidney function decline in hypertensive patients^18,19^. Additionally, urinary vanin-1 was shown to be significantly increased and proposed to be used as a biomarker in patients with obstructive nephropathy and IgA nephropathy^20,21^. However, no study has been done to evaluate urinary vanin-1 in the kidney transplantation setting to date. Therefore, in the current study, we aimed to assess the cross-sectional association of urinary vanin-1 with clinical and biochemical parameters including kidney function parameters and tubular injury markers, and to investigate the prospective association of urinary vanin-1 with graft failure in a large cohort of KTR with long-term follow-up.

## Method

### Study design and population

This study used data from TransplantLines Food and Nutrition Biobank and Cohort Study (NCT02811835). In this cohort, all adult KTR with a functioning graft of at least 1 year after transplantation without a history of malignancy or addiction who visited the transplant outpatient clinic at the University Medical Center Groningen (The Netherlands) between November 2008 and May 2011 were invited to participate. Of 817 eligible KTR, 707 were enrolled after providing written informed consent. For this study, KTR with missing 24 h urinary vanin-1 excretion measurement at baseline (n = 51) were excluded. The current study adhered to the Declarations of Helsinki and Istanbul and was approved by the Institutional Review Board of University Medical Center Groningen (METc 2008/186). This study was described following the Strengthening the Reporting of Observational Studies in Epidemiology (STROBE) guidelines (Supplementary Table 1)^22^.

The study end-point was death-censored graft failure, defined as the need for re-transplantation or (re-)initiation of dialysis. The end-point was recorded until December 2017. With the continuous surveillance system of the outpatient clinic of this university hospital, no patients were lost to follow-up.

### Data collection and laboratory measurements

The detailed protocol for data collection has been described previously^23^. In short, baseline clinical data were collected during a visit to the outpatient clinic. Relevant information regarding medication, donor, and transplantation was retrieved from the medical records. In the same outpatient visit, blood was drawn after an overnight fasting period. Furthermore, according to a strict protocol, all KTR were asked to collect a 24 h urine sample the day before their outpatient clinic visit.

Urinary vanin-1 concentration was measured by using enzyme-linked immunosorbent assay method (Human vanin-1 assay kit; cat. no. BI-VAN1U; Biomedica Immunoassays). There were 14 samples with urinary vanin-1 concentration values below the detection limit. For these, we gave a value of 0.075 pmol/L (i.e., half of the lowest measured urinary vanin-1 concentration in this study population) to allow for data transformation. To obtain 24 h urinary vanin-1 excretion value, urinary vanin-1 concentration was multiplied by 24 h urine volume. The eGFR was calculated using serum creatinine based on the 2009 chronic kidney disease epidemiology collaboration (CKD-EPI) equation^24^. Measurements of other tubular injury markers, i.e., urinary liver-type fatty acid-binding protein (L-FABP), urinary post-translationally modified fetuin-A protein (PTM-FetA), urinary epidermal growth factor (EGF), and plasma neutrophil gelatinase-associated lipocalin (NGAL), and tubular injury agents, i.e., urinary cooper and urinary soluble C5b-9 (sC5b-9), have been described in detail elsewhere^25–30^.

### Statistical analyses

For descriptive statistics, quantile–quantile plots were used to assess the normality of continuous variables and presented as mean ± standard deviation (SD) for normally distributed variables and as median [interquartile range (IQR)] for variables with a non-normal distribution. Categorical variables were expressed as numbers (valid percentages). Differences at baseline among subgroups of KTR according to the tertiles of 24 h urinary vanin-1 excretion were tested by one-way ANOVA for continuous variables with normal distribution, Kruskal–Wallis test for continuous variables with skewed distribution, and χ^2^ for categorical variables. Correlation between urinary vanin-1 excretion and kidney function parameters and other tubular injury markers were evaluated using Spearman’s rank correlation test.

The distribution of graft survival among subgroups of KTR based on the tertiles of 24 h urinary vanin-1 excretion was visualized using Kaplan–Meier curves, and the difference among curves was tested with a Log-rank test. Next, Cox proportional-hazard regression analyses were performed to assess dose–response by contrasting the risk of being in the second and third tertiles of 24 h urinary vanin-1 excretion against being in the first tertile for the development of death-censored graft failure, and by introducing 24 h urinary vanin-1 excretion as a continuous variable where it was log_2_-transformed to estimate the association with death-censored graft failure per doubling of 24 h urinary vanin-1 excretion. For these analyses, several adjustments were performed to account for the influence of potential confounders. In model 1, we adjusted for age, sex, and BSA. In model 2, we further adjusted for eGFR. In model 3, we further adjusted for log_2_ 24 h urinary protein excretion. In model 4 (full model), we further adjusted for the use of proliferation inhibitors. Schoenfeld residuals were tested, and the full models did not violate the assumption for proportionality of hazards (p = 0.7). Next to that, potential effect modification by age, sex, eGFR, and 24 h urinary protein excretion was tested by fitting both main effects and their cross-product terms in the full model, where p_interaction_ < 0.05 was considered to indicate effect modification.

In sensitivity analyses, we evaluated the association of 24 h urinary vanin-1 excretion with death-censored graft failure after excluding KTR with urinary vanin-1 value below the detection limit (n = 14). We repeated the analyses by excluding KTR with 24 h urinary vanin-1 excretion outside the 2.5th–97.5th percentile (n = 34) and outside the 5th–95th percentile (n = 66). In addition, a potential non-linear association of 24 h urinary vanin-1 excretion with death-censored graft failure was assessed by adding restricted cubic spline terms for vanin-1 to the models. The statistical significance of the improvement in model fit was assessed using the likelihood ratio test. Next, we repeated the Cox regression analyses where the 24 h urinary vanin-1 excretion variable was not transformed, transformed using the square root, and transformed using the inverse of each value. Finally, we repeated Cox regression analyses using urinary vanin-1 concentration and urinary vanin-1 concentration indexed for creatinine (urinary vanin-1/creatinine ratio) instead of 24 h urinary vanin-1 excretion.

For cross-sectional analyses, the original dataset was used, and variables with > 20 missing values were reported in the footnotes. For all prospective analyses, multiple imputations using Fully Conditional Specification were performed using the R package ‘mice’ (number of multiple imputations = 10) to account for missing data other than 24 h urinary vanin-1 excretion. All data analyses were performed with R version 4.0.5 (R Foundation for Statistical Computing, Vienna, Austria). A statistical significance level of p < 0.05 (two-tailed) was used for all analyses.

## Results

### Baseline characteristics

**Figure 1:**
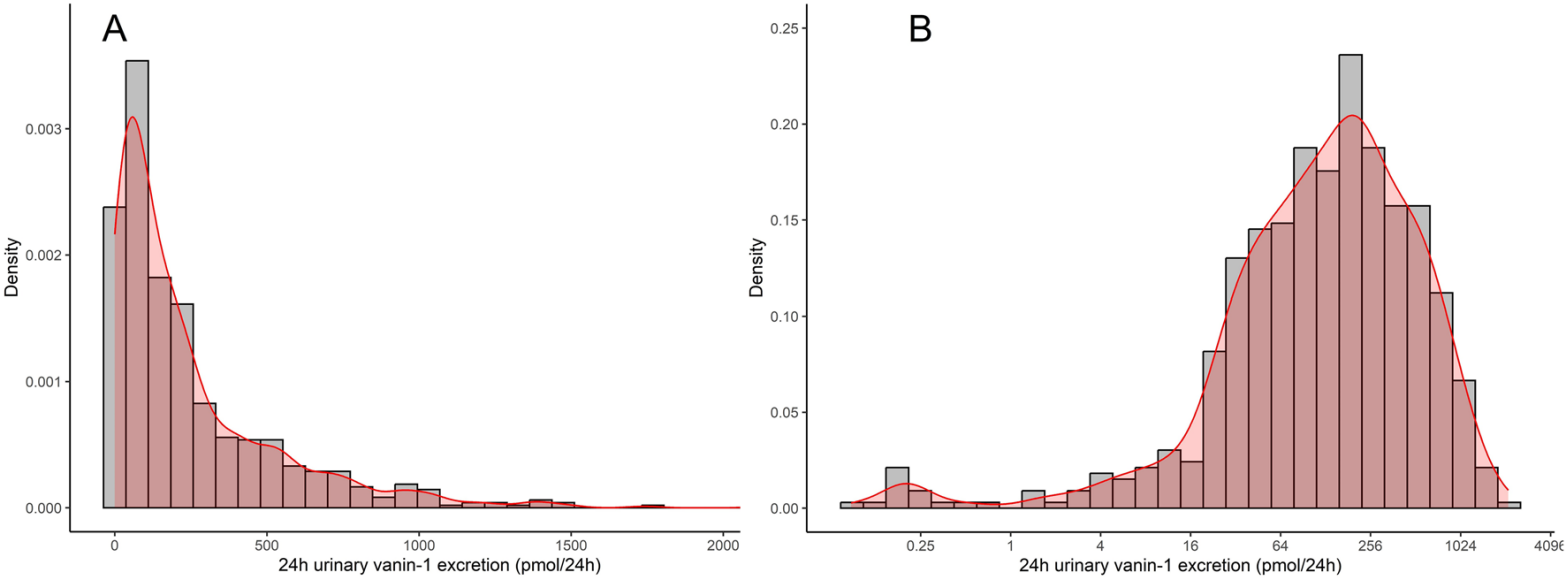
24 h urinary vanin-1 excretion distribution in the study population. (**A**) non-log-transformed; (**B**) log2-transformed.

The flow chart of the study population selection is presented in Supplementary Fig. 1. In total, 656 KTR (age 53 ± 13 years, 43% female, 5.4 [2–12] years after transplantation) were included in the analyses. Mean eGFR was 52.8 ± 20.5 mL/min/1.73 m^2^, and 24 h urinary albumin excretion was 38 [11–176] mg/24 h. The 24 h urinary vanin-1 excretion was 145 [51–331] pmol/24 h. The distribution of 24 h urinary vanin-1 excretion is presented in Fig. 1. When stratified based on the tertiles of 24 h urinary vanin-1 excretion, KTR in the highest tertile had the highest body surface area (BSA) (p = 0.035), highest urinary albumin excretion (p < 0.001), lowest use of proliferation inhibitors (p = 0.002), and lowest eGFR (p = 0.024). Furthermore, they also had the highest urinary sC5b-9 and urinary copper excretion (p = 0.002 and p < 0.001, respectively). However, there were no between-tertile differences in any of the other tubular injury markers (urinary L-FABP, urinary PTM-FetA, urinary EGF, and plasma NGAL) (Table 1).

**Table 1:** Baseline characteristics of the study participants.

Variables	Total N = 656	Tertile 1 N = 219 < 75 pmol/24 h	Tertile 2 N = 219 75–241.6 pmol/24 h	Tertile 3 N = 218 > 241.6 pmol/24 h	p-value
24 h urinary vanin-1 excretion, pmol/24 h	145 [51–331]	34 [18–51]	145 [104–192]	488 [332–699]	
Clinical characteristics					
Female sex, n (%)	283 (43.1)	104 (47.5)	95 (43.4)	84 (38.5)	0.2
Age, years	53 ± 13	51 ± 14	55 ± 12	54 ± 13	**0.007**
BMI, kg/m^2^	26.6 ± 4.8	26.5 ± 5.1	26.1 ± 4.6	27.2 ± 4.7	0.067
BSA, m^2^	1.94 ± 0.22	1.92 ± 0.23	1.92 ± 0.20	1.97 ± 0.22	**0.035**
SBP, mmHg	136 ± 18	136 ± 17	135 ± 17	138 ± 18	0.2
Diabetes, n (%)	158 (24.1)	52 (23.7)	60 (27.4)	46 (21.1)	0.3
Hypertension. n (%)	266 (40.7)	89 (41.0)	82 (37.4)	95 (43.8)	0.4
History of cardiovascular disease, n (%)	166 (25.3)	53 (24.2)	65 (29.7)	48 (22.0)	0.2
Current smoking, n (%)	76 (12.5)	25 (12.0)	20 (9.6)	31 (16.1)	0.1
Transplant-related characteristics					
First kidney transplant, n (%)	593 (90.5)	201 (92.2)	193 (88.1)	199 (91.3)	0.3
Pre-emptive transplant, n (%)	101 (15.4)	38 (17.4)	38 (17.4)	25 (11.5)	0.1
Time after transplantation, years	5.4 [2–12]	5.2 [2–10.2]	5.3 [1.6–12.1]	6.1 [2.34–13.7]	0.3
Donor age, years	43 ± 15	42 ± 15	44 ± 15	41 ± 16	0.2
Living donor, n (%)	220 (33.5)	70 (32.0)	76 (34.7)	74 (33.9)	0.8
Positive HLA class I antibodies, n (%)	98 (14.9)	34 (15.5)	34 (15.5)	30 (13.8)	0.8
Positive HLA class II antibodies, n (%)	111 (16.9)	41 (18.7)	30 (13.7)	40 (18.3)	0.3
Cold ischemia time, hours	15.5 [2.9–21.3]	15.5 [3–22]	15.6 [2.8–21]	15.1 [2.7–21.7]	0.5
History of delayed graft function, n (%)	48 (7.3)	14 (6.4)	18 (8.2)	16 (7.3)	0.8
History of rejection, n (%)	173 (26.4)	48 (21.9)	61 (27.9)	64 (29.4)	0.2
Immunosuppressive medication					
Daily prednisolone dose, mg/24 h	10.0 [7.5–10.0]	10.0 [7.5–10.0]	10.0 [7.5–10.0]	10.0 [7.5–10.0]	0.5
Calcineurin inhibitor, n (%)	378 (57.6)	112 (51.1)	132 (60.3)	134 (61.5)	0.057
Proliferation inhibitor, n (%)	543 (82.8)	192 (87.7)	186 (84.9)	165 (75.7)	**0.002**
mTOR inhibitor, n (%)	22 (3.4)	8 (3.7)	6 (2.7)	8 (3.7)	0.8
Laboratory measurements					
Hb, mmol/L	8.2 ± 1.1	8.2 ± 1.1	8.2 ± 1.1	8.3 ± 1.1	0.4
HbA1c, %	5.8 [5.5–6.2]	5.7 [5.5–6.2]	5.8 [5.5–6.3]	5.8 [5.5–6.2]	0.3
Total cholesterol, mmol/L	5.12 ± 1.14	5.11 ± 1.15	5.03 ± 1.17	5.23 ± 1.08	0.2
LDL cholesterol, mmol/L	2.98 ± 0.94	3.01 ± 0.97	2.87 ± 0.94	3.07 ± 0.90	0.081
HDL cholesterol, mmol/L	1.3 [1.1–1.6]	1.3 [1.1–1.6]	1.3 [1.1–1.7]	1.3 [1.0–1.7]	0.7
Triglycerides, mmol/L	1.68 [1.25–2.28]	1.67 [1.15–2.26]	1.62 [1.24–2.24]	1.76 [1.29–2.33]	0.2
Serum ferritin, µg/mL	121 [55–226]	110 [56–201]	109 [52–235]	129 [59–229]	0.4
TSAT, %	25.2 ± 10.7	24.8 ± 11	24.8 ± 10.9	25.9 ± 10.3	0.5
hs-CRP, mg/L	1.6 [0.7–4.7]	1.6 [0.8–5.3]	1.5 [0.6–4.6]	1.7 [0.8–4.5]	0.6
Kidney function and glomerular injury parameters					
Serum creatinine, µmol/L	123 [99–160]	117 [96–148]	126 [101–164]	128 [105–164]	**0.030**
eGFR, mL/min/1.73 m^2^	52.8 ± 20.5	55.8 ± 21.7	51 ± 19.8	51.4 ± 19.8	**0.024**
Urinary albumin excretion, mg/24 h	38 [11–176]	27 [8–111]	43 [10–161]	58 [13–386]	** < 0.001**
Urinary protein excretion, g/24 h	0.21 [0.01–0.37]	0.16 [0.01–0.29]	0.21 [0.01–0.35]	0.24 [0.01–0.64]	** < 0.001**
Tubular injury markers					
Urinary L-FABP excretion, µg/24 h	2.07 [0.90–7.07]	1.88 [0.85–5.34]	2.06 [0.84–9.15]	2.61 [1.08–7.99]	0.055
Urinary PTM-FetA excretion, µg/24 h	32.9 [17.1–73.2]	26.4 [14.1–62.5]	34.8 [18.6–69.3]	37.4 [18.4–89.4]	0.057
Urinary EGF excretion, µg/24 h	9.43 [4.71–19.3]	10.41 [5.63–21.46]	8.71 [4.22–16.34]	9.59 [4.61–17.53]	0.2
Plasma NGAL, µg/L	170 [132–232]	162 [126–228]	166 [132–223]	180 [137–242]	0.2
Tubular injury agents					
Urinary sC5b-9 excretion, µg/24 h	5.09 [4.02–6.53]	4.84 [3.77–5.9]	5.11 [3.96–6.18]	5.28 [4.37–8.15]	**0.002**
Urinary copper excretion, µg/24 h	23.2 [15.6–35.3]	21.9 [14.8–31.7]	21.5 [14.8–33.3]	25.4 [18.4–40.8]	** < 0.001**

Smoking status was missing in 47 (7.2%) patients, HbA1C was missing in 29 (4.4%) patients, hs-CRP was missing in 41 (6.2%) patients, urinary L-FABP was missing in 40 (6.1%) patients, urinary EGF was missing in 51 (7.8%) patients, and urinary PTM-FetA and urinary sC5b-9 was missing in 62 (9.5%) patients.

BMI, body mass index; BSA, body surface area; EGF, epidermal growth factor; eGFR, estimated glomerular filtration rate according to creatinine-based CKD-EPI formula; HbA1c, hemoglobin A1c; hs-CRP, high-sensitivity C-reactive protein; HLA, human leucocyte antigen; L-FABP, liver type-fatty acid binding proteins; mTOR, mechanistic target of rapamycin; NGAL, Neutrophil gelatinase-associated lipocalin; PTM-FetA, post-translationally modified fetuin-A protein; SBP, systolic blood pressure; sC5b-9, soluble C5b-9; TSAT, transferrin saturation.

Significant values are in bold.

### Correlation of urinary vanin-1 with markers of tubular injury and kidney function parameters

**Figure 2:**
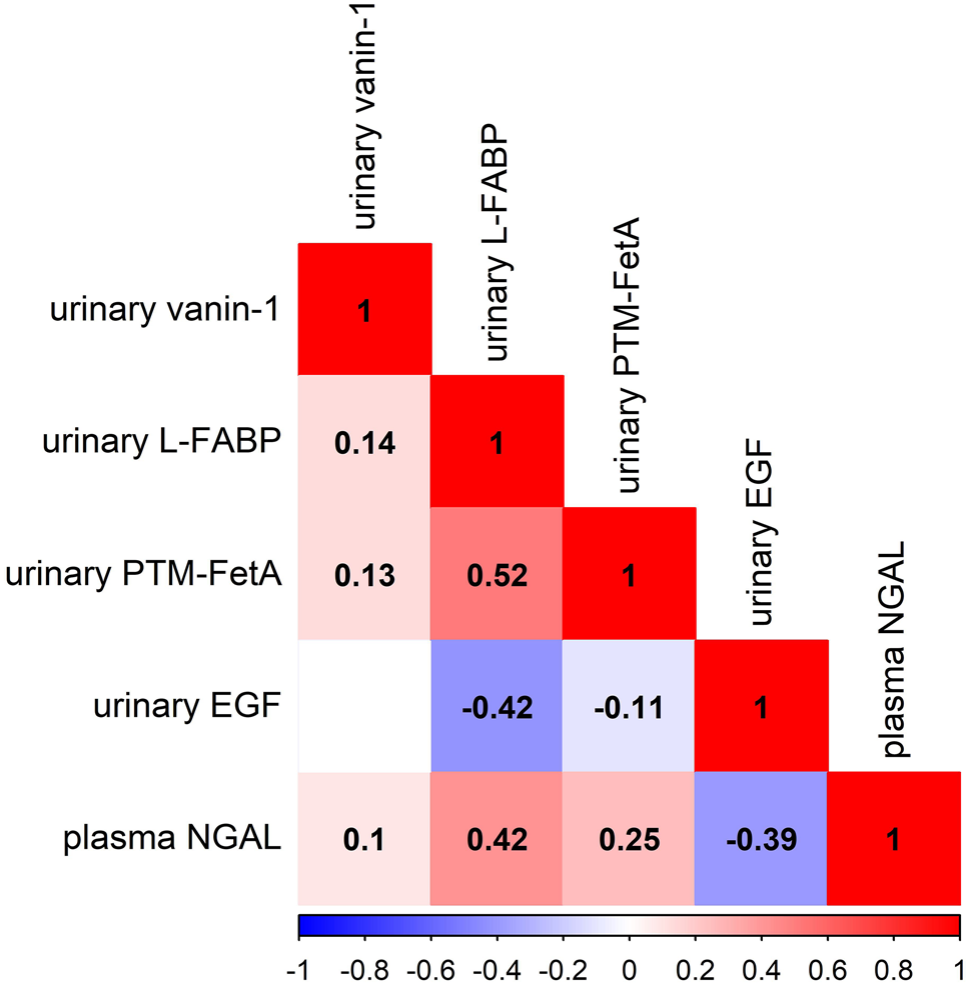
Correlation between urinary vanin-1 and tubular injury markers. Spearman rank correlation test was used. Values indicated the correlation coefficient. Blank value indicated a non-significant correlation (p > 0.05). EGF, epidermal growth factor; eGFR, estimated glomerular filtration rate; L-FABP, liver-type fatty acid binding protein; NGAL, Neutrophil gelatinase-associated lipocalin; PTM-FetA, post-translationally modified fetuin-A protein.

**Figure 3:**
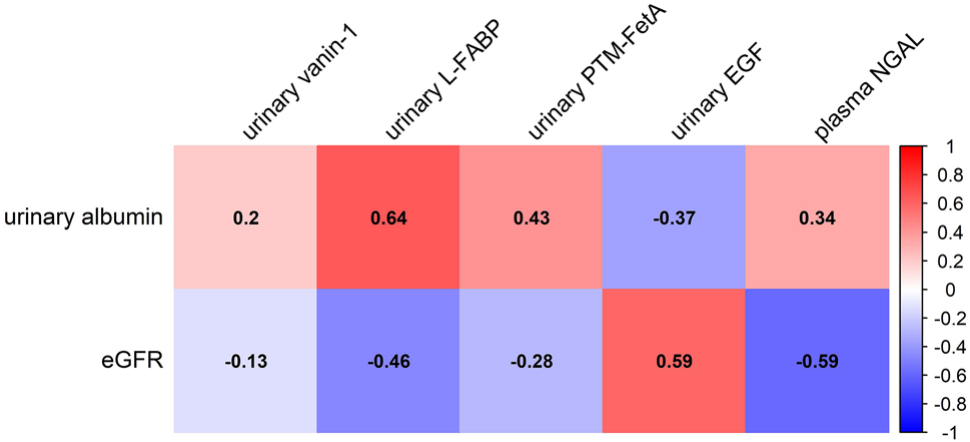
Correlation between kidney function and urinary albumin and urinary vanin-1 and other tubular injury markers. Spearman rank correlation test was used. Values indicated the correlation coefficient. Blank value indicated a non-significant correlation (p > 0.05). EGF, epidermal growth factor; eGFR, estimated glomerular filtration rate; L-FABP, liver-type fatty acid binding protein; NGAL, Neutrophil gelatinase-associated lipocalin; PTM-FetA, post-translationally modified fetuin-A protein.

Urinary vanin-1 correlated significantly with urinary L-FABP (ρ = 0.14, p < 0.001), urinary PTM-FetA (ρ = 0.13, p = 0.001), and plasma NGAL (ρ = 0.10, p = 0.011), but not with urinary EGF (ρ = − 0.05, p = 0.3). However, other tubular injury markers had stronger correlation coefficients with each other (Fig. 2). Urinary vanin-1 was also positively correlated with urinary albumin (ρ = 0.20, p < 0.001) and inversely correlated with eGFR (ρ = − 0.13, p = 0.001). Nevertheless, other tubular injury markers had a stronger correlation with urinary albumin and eGFR (Fig. 3).

### Urinary vanin-1 and graft failure

**Figure 4:**
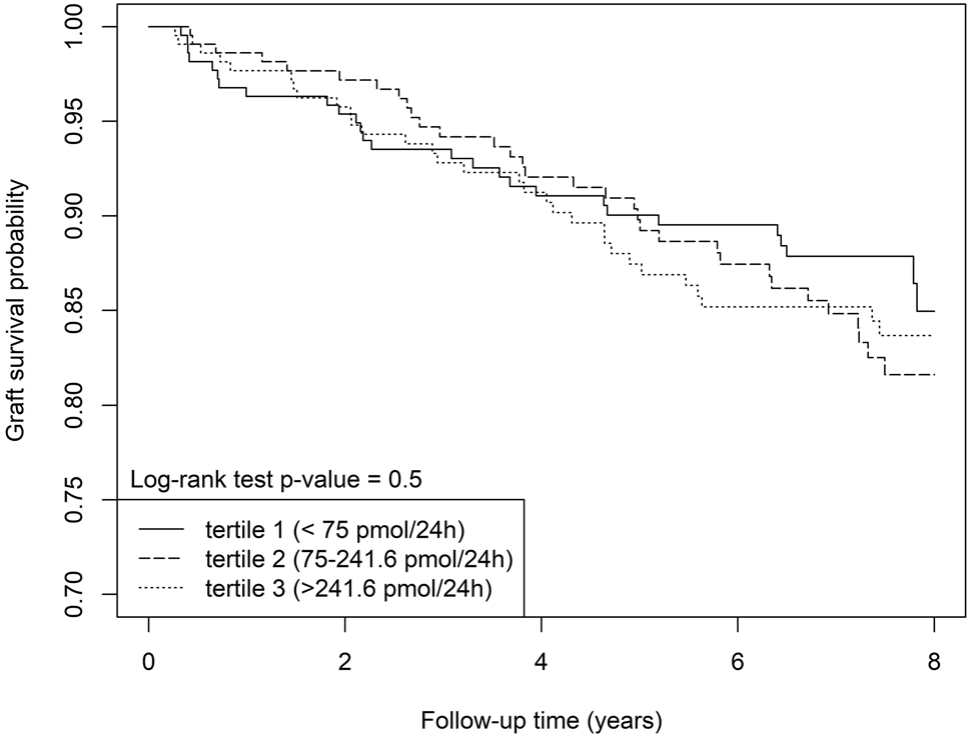
Kaplan–Meier analysis for death-censored graft survival per tertile of 24 h urinary vanin-1 excretion.

During a follow-up of 7.4 [4.9–8.0] years, 94 (14.3%) KTR developed death-censored graft failure. Death-censored graft failure occurred in 27 (12.3%), 35 (16%), and 32 (14.7%) patients in the first, second, and third tertile of 24 h urinary vanin-1 excretion, respectively (p_log-rank_ = 0.5) (Fig. 4). Univariable and multivariable Cox proportional-hazard models showed no prospective associations between urinary vanin-1 excretion and death-censored graft failure when presented as continuous variables or as tertiles (Table 2). There was no indication of effect-modification by age, sex, eGFR, or urinary protein excretion for the association between 24 h urinary vanin-1 excretion and death-censored graft failure.

**Table 2:** Prospective analysis of the association of 24 h urinary vanin-1 excretion with death-censored graft failure in 656 kidney transplant recipients.

	Tertile 1 N = 219 < 75 pmol/24 h	Tertile 2 N = 219 75–241.6 pmol/24 h		Tertile 3 N = 218 > 241.6 pmol/24 h		Continuous (per doubling)	
n_events_	27	35		32		94	
Model		HR (95%CI)	p-value	HR (95%CI)	p-value	HR (95%CI)	p-value
Crude	Ref	1.38 (0.83–2.27)	0.2	1.18 (0.70–1.98)	0.5	1.09 (0.99–1.20)	0.093
Model 1	Ref	1.47 (0.88–2.43)	0.1	1.23 (0.73–2.06)	0.4	1.09 (0.99–1.20)	0.073
Model 2	Ref	1.10 (0.66–1.83)	0.7	1.00 (0.60–1.68)	1.0	1.04 (0.93–1.16)	0.5
Model 3	Ref	0.90 (0.54–1.51)	0.7	0.72 (0.42–1.23)	0.2	0.96 (0.86–1.08)	0.5
Model 4	Ref	0.89 (0.53–1.50)	0.7	0.71 (0.41–1.21)	0.2	0.96 (0.86–1.07)	0.5

Cox proportional-hazard regression analyses were performed to assess the association of 24h urinary vanin-1 excretion with the risk of death-censored graft failure (the need for re-transplantation or (re-)initiation of dialysis). Model 1 was adjusted for age, sex, and body surface area. Model 2 was further adjusted for the estimated glomerular filtration rate based on the creatinine-based CKD-EPI formula. Model 3 was further adjusted for 24-h urinary protein excretion. Model 4 was further adjusted for the use of proliferation inhibitors. 95% CI, 95% confidence interval; HR, hazard ratio.

### Sensitivity analyses

After excluding 14 KTR with urinary vanin-1 level below the detection limit, the association of 24 h urinary vanin-1 excretion with death-censored graft failure remained unchanged (Supplementary Table 2). Similarly, excluding 34 KTR with 24 h urinary vanin-1 excretion outside 2.5th–97.5th percentile (i.e., < 1.64 pmol/24 h and > 1041 pmol/24 h) or 66 KTR with 24 h urinary vanin-1 excretion outside 5th–95th percentile (i.e., < 6.87 pmol/24 h and > 840 pmol/24 h) also did not materially change the association with death-censored graft failure (Supplementary Tables 3 and 4). The model including spline terms for urinary vanin-1 excretion did not improve the model fit for death-censored graft failure compared to the model without spline terms (p_likelihood ratio_ = 0.8), and the spline term was not associated with death-censored graft failure (p = 0.8). Additional sensitivity analyses where urinary vanin-1 excretion was transformed with several different transformations also showed no significant associations between urinary vanin-1 excretion and death-censored graft failure (Supplementary Table 5). Final sensitivity analyses where urinary vanin-1 concentration and urinary vanin-1/creatinine ratio were used instead of 24 h urinary vanin-1 excretion showed that urinary vanin-1 was associated with death-censored graft failure in the unadjusted model. However, the association was lost after further adjustment for eGFR (Supplementary Tables 6 and 7).

## Discussion

In this large cohort of KTR, we aimed to assess the association of 24 h urinary vanin-1 excretion with clinical and biochemical parameters including kidney function parameters and tubular injury markers, and to investigate the prospective association of urinary vanin-1 with graft failure. We found that eGFR was lower and 24 h urinary albumin excretion was higher across the increasing tertiles of 24 h urinary vanin-1 excretion. Next to that, 24 h urinary sC5b-9 excretion and 24 h urinary copper excretion as respective markers of tubular complement activation and enhancer of oxidative stress that cause tubular injury^29,30^ were also higher across the increasing tertiles of 24 h urinary vanin-1 excretion. 24 h urinary vanin-1 excretion significantly correlated with most of the tubular injury markers; however, other tubular injury markers showed stronger correlations among each other. Furthermore, compared to other tubular injury markers, 24 h urinary vanin-1 excretion had the weakest correlation with kidney function parameters. In prospective analyses, 24 h urinary vanin-1 excretion was not independently associated with death-censored graft failure.

In general, the presence of molecules in the urine might have originated from the circulation, from the local production by the kidney, or a combination of both. Since vanin-1 is a glycosylphosphatidylinositol membrane-bound molecule that is expressed by the proximal tubule epithelial cells^11,12, 31^, a possible source of vanin-1 in the urine is shedding. Findings from experimental studies using different rat models indicated that the increased urinary vanin-1 level originates from the shedding of vanin-1 from proximal tubule epithelial cells^12,16, 32^.

A previous observational study in patients with hypertension reported that urinary vanin-1 correlated positively with urinary albumin and inversely with eGFR^18^. This aligns with our findings, although the correlation coefficient with albumin and eGFR in our study was weaker than in the previous study^18^. Next to that, previous experimental and observational studies compared urinary vanin-1 with urinary kidney injury molecule-1 (KIM-1), urinary N-acetyl-β-d-glucosaminidase (NAG), and urinary NGAL^12,16–18, 20, 33^. While these biomarkers were not available in our cohort, we did have data on urinary L-FABP, PTM-FetA, EGF, and plasma NGAL, which have been previously associated with death-censored graft failure^25–28^. Urinary vanin-1 correlated with all tubular injury biomarkers except for urinary EGF. This might be explained by differences in the location of vanin-1 and EGF expression in the tubules. While vanin-1 is expressed in the proximal tubule^11,12^, EGF is expressed in the limb of Henle and the distal tubule^27^.

The urinary vanin-1 level increases early upon tubular injury and decreases when the tubules are already severely injured^12,16^. Thus, we posit that the timing of the urinary vanin-1 measurements might be of importance and might explain the weak correlation between urinary vanin-1 and other tubular injury markers, as well as the absence in the association with death-censored graft failure. To investigate this, simultaneous evaluation of the degree of tubular injury from the kidney biopsy and measurements of urinary vanin-1 and other tubular injury markers would be required. However, KTR in this cohort did not structurally undergo a kidney biopsy procedure when the blood and urine were collected.

There are several important limitations in this study. First, this was a single-center study with an over-representation of the Caucasian population. Thus, the present findings cannot be generalized to other populations with different ethnicities. Second, as this was an observational study, residual confounding may still exist despite the number of potentially confounding factors that we have adjusted for. Third, we did not have biopsy data available at baseline to allow for a correlation with structural kidney damage. Fourth, urinary vanin-1 excretion was assessed at a single time point at baseline. We did not have repeated urine collections that allowed for multiple measurements of urinary vanin-1 excretion, by which we could have accounted for the dynamics of urinary vanin-1 excretion. Future studies with sequential analyses of urinary vanin-1 excretion might be an option to confirm the findings in this study. Lastly, urinary vanin-1 excretion was measured at different times after kidney transplantation. While this may be seen as a study limitation, the justification for this is that we wanted to evaluate the association of the biomarker with graft failure in the real-world outpatient clinical setting, in which patients visit the outpatient clinic at different times after kidney transplantation instead of only at specific time points (e.g., at 6 or 12 months after transplantation).

In conclusion, this is the first study to evaluate 24 h urinary vanin-1 excretion in a study population of stable, outpatient KTR. Our findings do not support 24 h urinary vanin-1 excretion as a useful biomarker to assess the risk of death-censored graft failure.

### Supplementary Information

Supplementary Information.

## Data Availability

Public sharing of individual participant data was not included in the informed consent of the TransplantLines Biobank and Cohort Study, but data can be made available to interested researchers upon reasonable request by sending an e-mail to the data manager of the TransplantLines Biobank and Cohort study (datarequest.transplantlines@umcg.nl).
