# Supplementary material for: Urinary vanin-1, tubular injury, and graft failure in kidney transplant recipients
Source: Sci Rep. 2024 Jan 27;14:2283. doi: 10.1038/s41598-024-52635-x (PMC10821939; doi:10.1038/s41598-024-52635-x)
Supplement: Supplementary file 1 — Supplementary Information. [file 41598_2024_52635_MOESM1_ESM.pdf]

Urinary Vanin-1, Tubular Injury, and Graft Failure in Kidney Transplant Recipients

SUPPLEMENTAL MATERIAL

Table of contents

**Supplementary Figure 1.** Flow chart of the study population selection	Page 2
**Supplementary Table 1.** STROBE Statement – Checklist of items that should be included in reports of cohort studies.	Page 3
**Supplementary Table 2.** Prospective analysis of the association of 24h urinary vanin-1 excretion with death-censored graft failure in which urinary vanin-1 below the detection limit was excluded.	Page 6
**Supplementary Table 3.** Prospective analysis of the association of 24h urinary vanin-1 excretion with death-censored graft failure in which 24h urinary vanin-1 excretion outside the 2.5th-97.5th percentile was excluded.	Page 7
**Supplementary Table 4.** Prospective analysis of the association of 24h urinary vanin-1 excretion with death-censored graft failure in which 24h urinary vanin-1 excretion outside the 5th-95th percentile was excluded.	Page 8
**Supplementary Table 5.** Cox proportional-hazard regression analysis of the associations of urinary vanin-1 excretion with graft failure using various transformations in adjusted Cox regression models.	Page 9
**Supplementary Table 6.** Prospective analysis of the association of urinary Vanin-1 concentration with death-censored graft failure in 656 kidney transplant recipients.	Page 10
**Supplementary Table 7.** Prospective analysis of the association of urinary Vanin-1/creatinine ratio with death-censored graft failure in 656 kidney transplant recipients.	Page 11

Supplementary Figure 1. Flow chart of the study population selection

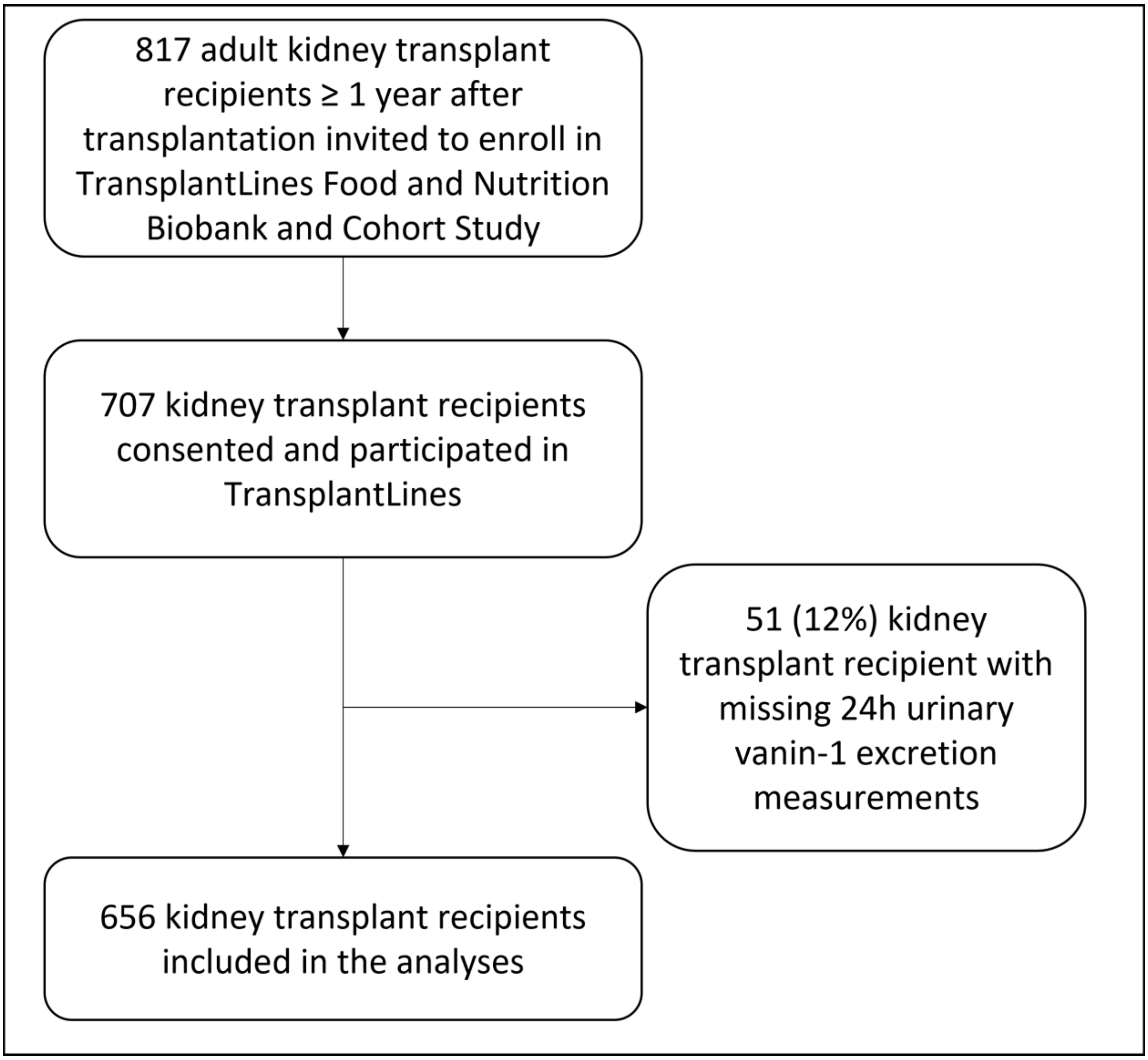

Supplementary Table 1. STROBE Statement – Checklist of items that should be included in reports of cohort studies.

	Item No	Recommendation	Page No
**Title and abstract**	1	(*a*) Indicate the study's design with a commonly used term in the title or the abstract	Title and Abstract
		(*b*) Provide in the abstract an informative and balanced summary of what was done and what was found	Abstract
**Introduction**			
Background/rationale	2	Explain the scientific background and rationale for the investigation being reported	Introduction par 1-4
Objectives	3	State specific objectives, including any prespecified hypotheses	Introduction par 4
**Methods**			
Study design	4	Present key elements of study design early in the paper	Methods par 1
Setting	5	Describe the setting, locations, and relevant dates, including periods of recruitment, exposure, follow-up, and data collection	Methods par 1-3
Participants	6	(*a*) Give the eligibility criteria, and the sources and methods of selection of participants. Describe methods of follow-up	Methods par 1 and 2
		(*b*) For matched studies, give matching criteria and number of exposed and unexposed	N/A
Variables	7	Clearly define all outcomes, exposures, predictors, potential confounders, and effect modifiers. Give diagnostic criteria, if applicable	Methods par 2 and 6
Data sources/ measurement	8*	For each variable of interest, give sources of data and details of methods of assessment (measurement). Describe comparability of assessment methods if there is more than one group	Methods par 3 and 4
Bias	9	Describe any efforts to address potential sources of bias	Methods par 5
Study size	10	Explain how the study size was arrived at	Supplementary figure 1
Quantitative variables	11	Explain how quantitative variables were handled in the analyses. If applicable, describe which groupings were chosen and why	Methods par 5
Statistical methods	12	(*a*) Describe all statistical methods, including those used to control for confounding	Methods par 5-7
		(*b*) Describe any methods used to examine subgroups and interactions	Methods par 6

		(*c*) Explain how missing data were addressed	Methods par 8
		(*d*) If applicable, explain how loss to follow-up was addressed	N/A
		(*e*) Describe any sensitivity analyses	Methods par 7
**Results**			
Participants	13*	(a) Report numbers of individuals at each stage of study—eg numbers potentially eligible, examined for eligibility, confirmed eligible, included in the study, completing follow-up, and analysed	Supplementary Figure 1
		(b) Give reasons for non-participation at each stage	Supplementary Figure 1
		(c) Consider use of a flow diagram	Supplementary Figure 1
Descriptive data	14*	(a) Give characteristics of study participants (eg demographic, clinical, social) and information on exposures and potential confounders	Results par 1<br>Table 1
		(b) Indicate number of participants with missing data for each variable of interest	Footnotes tables
		(c) Summarise follow-up time (eg, average and total amount)	Results par 3
Outcome data	15*	Report numbers of outcome events or summary measures over time	Results par 3
Main results	16	(*a*) Give unadjusted estimates and, if applicable, confounder-adjusted estimates and their precision (eg, 95% confidence interval). Make clear which confounders were adjusted for and why they were included	Table 2, Supplementary Table 3-7
		(*b*) Report category boundaries when continuous variables were categorized	Table 2, Supplementary Table 3, 4, 6, 7
		(*c*) If relevant, consider translating estimates of relative risk into absolute risk for a meaningful time period	N/A
Other analyses	17	Report other analyses done—eg analyses of subgroups and interactions, and sensitivity analyses	Results par 4

**Discussion**			
Key results	18	Summarise key results with reference to study objectives	Discussion par 1
Limitations	19	Discuss limitations of the study, taking into account sources of potential bias or imprecision. Discuss both direction and magnitude of any potential bias	Discussion par 5
Interpretation	20	Give a cautious overall interpretation of results considering objectives, limitations, multiplicity of analyses, results from similar studies, and other relevant evidence	Discussion par 2-5
Generalisability	21	Discuss the generalisability (external validity) of the study results	Discussion par 5
**Other information**			
Funding	22	Give the source of funding and the role of the funders for the present study and, if applicable, for the original study on which the present article is based	Funding section

Supplementary Table 2. Prospective analysis of the association of 24h urinary vanin-1 excretion with death-censored graft failure in which urinary vanin-1 below the detection limit were excluded.

	Tertile 1<br>N = 214<br>< 82.8<br>pmol/24h	Tertile 2<br>N = 214<br>83 – 247 pmol/24h		Tertile 3<br>N = 214<br>> 247 pmol/24h		Continuous (Per doubling)	
n events	26	35		32		93	
Model		HR (95%CI)	p-value	HR (95%CI)	p-value	HR (95%CI)	p-value
Crude	Ref	1.42 (0.85-2.35)	0.2	1.21 (0.72-2.04)	0.5	1.08 (0.93-0.97)	0.2
Model 1	Ref	1.49 (0.89-2.49)	0.1	1.25 (0.74-2.10)	0.4	1.09 (0.97-1.21)	0.1
Model 2	Ref	1.13 (0.67-1.89)	0.6	1.05 (0.62-1.77)	0.9	1.06 (0.94-1.19)	0.4
Model 3	Ref	0.91 (0.54-1.53)	0.7	0.75 (0.44-1.29)	0.3	0.97 (0.85-1.10)	0.6
Model 4	Ref	0.90 (0.53-1.52)	0.7	0.74 (0.43-1.27)	0.3	0.98 (0.87-1.12)	0.8

Of 656 kidney transplant recipients, 14 of them had urinary vanin-1 below the detection limit, leaving 642 kidney transplant recipients for Cox proportional-hazard regression analyses. Death-censored graft failure was defined as the need for re-transplantation or (re-)initiation of dialysis. Model 1 was adjusted for age, sex, and body surface area. Model 2 was further adjusted for the estimated glomerular filtration rate based on the creatinine-based CKD-EPI formula. Model 3 was further adjusted for 24-hour urinary protein excretion. Model 4 was further adjusted for the use of proliferation inhibitors. 95% CI, 95% confidence interval; HR, hazard ratio.

Supplementary Table 3. Prospective analysis of the association of 24h urinary vanin-1 excretion with death-censored graft failure in which 24h urinary vanin-1 excretion outside the 2.5th-97.5th percentile were excluded.

	Tertile 1<br>N = 208<br>< 79 pmol/24h	Tertile 2<br>N = 207<br>79 – 239 pmol/24h		Tertile 3<br>N = 207<br>> 239 pmol/24h		Continuous (Per doubling)	
n_{events}	26	33		31		90	
Model		HR (95%CI)	p-value	HR (95%CI)	p-value	HR (95%CI)	p-value
Crude	Ref	1.34 (0.80-2.24)	0.3	1.17 (0.69-1.98)	0.6	1.08 (0.96-1.22)	0.2
Model 1	Ref	1.43 (0.85-2.40)	0.2	1.21 (0.71-2.05)	0.5	1.09 (0.96-1.22)	0.2
Model 2	Ref	1.10 (0.65-1.85)	0.7	1.01 (0.60-1.71)	1.0	1.04 (0.92-1.18)	0.6
Model 3	Ref	0.92 (0.54-1.57)	0.8	0.76 (0.44-1.30)	0.3	0.96 (0.84-1.10)	0.6
Model 4	Ref	0.91 (0.54-1.56)	0.7	0.74 (0.43-1.28)	0.3	0.98 (0.86-1.12)	0.8

Of 656 kidney transplant recipients, 17 of them had urinary vanin-1 below the 2.5th percentile, and another 17 had urinary vanin-1 above the 97.5th percentile, leaving 622 kidney transplant recipients for Cox proportional-hazard regression analyses. Death-censored graft failure was defined as the need for re-transplantation or (re-)initiation of dialysis. Model 1 was adjusted for age, sex, and body surface area. Model 2 was further adjusted for the estimated glomerular filtration rate based on the creatinine-based CKD-EPI formula. Model 3 was further adjusted for 24-hour urinary protein excretion. Model 4 was further adjusted for the use of proliferation inhibitors. 95% CI, 95% confidence interval; HR, hazard ratio.

Supplementary Table 4. Prospective analysis of the association of 24h urinary vanin-1 excretion with death-censored graft failure in which 24h urinary vanin-1 excretion outside the 5th-95th percentile were excluded.

	Tertile 1<br>N = 197<br>< 84.3<br>pmol/24h	Tertile 2<br>N = 197<br>84.3 – 230 pmol/24h		Tertile 3<br>N = 196<br>> 230 pmol/24h		Continuous (Per doubling)	
n_{events}	25	33		27		85	
Model		HR (95%CI)	p-value	HR (95%CI)	p-value	HR (95%CI)	p-value
Crude	Ref	1.38 (0.82-2.32)	0.2	1.06 (0.61-1.83)	0.8	1.05 (0.92-1.21)	0.5
Model 1	Ref	1.47 (0.87-2.47)	0..2	1.10 (0.63-1.90)	0.7	1.06 (-.93-1.21)	0.4
Model 2	Ref	1.10 (0.65-1.87)	0.7	0.90 (1.11-1.56)	0.7	1.00 (0.87-1.15)	1.0
Model 3	Ref	0.92 (0.54-1.58)	0.8	0.68 (0.39-1.19)	0.2	0.93 (0.80-1.07)	0.3
Model 4	Ref	0.92 (0.54-1.57)	0.7	0.67 (0.38-1.19)	0.2	0.95 (0.82-1.10)	0.5

Of 656 kidney transplant recipients, 33 of them had urinary vanin-1 below the 5th percentile, and another 33 had urinary vanin-1 above the 95th percentile, leaving 590 kidney transplant recipients for Cox proportional-hazard regression analyses. Death-censored graft failure was defined as the need for re-transplantation or (re-)initiation of dialysis. Model 1 was adjusted for age, sex, and body surface area. Model 2 was further adjusted for the estimated glomerular filtration rate based on the creatinine-based CKD-EPI formula. Model 3 was further adjusted for 24-hour urinary protein excretion. Model 4 was further adjusted for the use of proliferation inhibitors. 95% CI, 95% confidence interval; HR, hazard ratio.

Supplementary Table 5. Cox proportional-hazard regression analysis of the associations of urinary vanin-1 excretion with graft failure using various transformations in adjusted Cox regression models.

Transformation	Graft failure n_{event} = 79/656	
	HR (95% CI)	p-value
Standardized urinary vanin-1 excretion	1.00 (1.00-1.00)	0.7
Square root urinary vanin-1 excretion	0.99 (0.96-1.02)	0.5
1/Urinary vanin-1 excretion	1.24 (0.62-2.47)	0.5

Models were adjusted for age, sex, body surface area, estimated glomerular filtration rate based on creatinine-based CKD-EPI formula, 24-hour urinary protein excretion, and the use of proliferation inhibitor. 95% CI, 95% confidence interval; HR, hazard ratio.

Supplementary Table 6. Prospective analysis of the association of urinary Vanin-1 concentration with death-censored graft failure in 656 kidney transplant recipients.

	Tertile 1<br>N = 219<br>< 32.3 pmol/L	Tertile 2<br>N = 219<br>32.3 – 109.3 pmol/L		Tertile 3<br>N = 218<br>> 109.3 pmol/L		Continuous (Per doubling)	
n $_{events}$	23	37		34		94	
Model		HR (95%CI)	p-value	HR (95%CI)	p-value	HR (95%CI)	p-value
Crude	Ref	1.73 (1.02-2.90)	0.040	1.50 (0.88-2.56)	0.1	1.11 (1.00-1.22)	0.044
Model 1	Ref	1.85 (1.09-3.13)	0.022	1.53 (0.89-2.61)	0.1	1.11 (1.01-1.22)	0.039
Model 2	Ref	1.33 (0.78-2.25)	0.3	1.16 (0.68-1.98)	0.6	1.05 (0.94-1.18)	0.4
Model 3	Ref	1.09 (0.63-1.87)	0.8	0.85 (0.49-1.47)	0.6	0.98 (0.87-1.09)	0.7
Model 4	Ref	0.98 (0.54-1.75)	0.9	0.71 (0.39-1.30)	0.3	0.97 (0.87-1.09)	0.6

Cox proportional-hazard regression analyses were performed to assess the association of urinary vanin-1 concentration with the risk of death-censored graft failure (the need for re-transplantation or (re-)initiation of dialysis). Model 1 was adjusted for age, sex, and body surface area. Model 2 was further adjusted for the estimated glomerular filtration rate based on the creatinine-based CKD-EPI formula. Model 3 was further adjusted for 24-hour urinary protein excretion. Model 4 was further adjusted for the use of proliferation inhibitors. 95% CI, 95% confidence interval; HR, hazard ratio.

Supplementary Table 7. Prospective analysis of the association of urinary Vanin-1/creatinine ratio with death-censored graft failure in 656 kidney transplant recipients.

	Tertile 1<br>N = 219<br>< 7.21<br>pmol/mmol	Tertile 2<br>N = 219<br>7.21 – 21.78 pmol/mmol		Tertile 3<br>N = 218<br>> 21.78 pmol/mmol		Continuous (Per doubling)	
n_{events}	21	41		32		94	
Model		HR (95%CI)	p-value	HR (95%CI)	p-value	HR (95%CI)	p-value
Crude	Ref	2.11 (1.25-3.57)	0.005	1.65 (0.95-2.86)	0.078	1.11 (1.01-1.23)	0.039
Model 1	Ref	2.24 (1.32-3.81)	0.003	1.74 (1.00-3.03)	0.051	1.12 (1.01-1.24)	0.025
Model 2	Ref	1.53 (0.89-2.60)	0.1	1.44 (0.82-2.52)	0.2	1.07 (0.95-1.20)	0.3
Model 3	Ref	1.19 (0.69-2.05)	0.5	1.03 (0.58-1.83)	0.9	0.99 (0.88-1.11)	0.8
Model 4	Ref	1.11 (0.61-2.03)	0.7	0.96 (0.51-1.83)	0.9	0.99 (0.88-1.11)	0.8

Cox proportional-hazard regression analyses were performed to assess the association of urinary vanin-1/creatinine ratio with the risk of death-censored graft failure (the need for re-transplantation or (re-)initiation of dialysis). Model 1 was adjusted for age, sex, and body surface area. Model 2 was further adjusted for the estimated glomerular filtration rate based on the creatinine-based CKD-EPI formula. Model 3 was further adjusted for 24-hour urinary protein excretion. Model 4 was further adjusted for the use of proliferation inhibitors. 95% CI, 95% confidence interval; HR, hazard ratio.
